# Metabolomics-based study of potential biomarkers of sepsis

**DOI:** 10.1038/s41598-022-24878-z

**Published:** 2023-01-11

**Authors:** Yang Li, Chenglin Wang, Muhu Chen

**Affiliations:** Department of Emergency Medicine, The Affiliated Hospital of Southwest Medical University, Luzhou, 646000 Sichuan China

**Keywords:** Infection, Diseases, Immunological disorders

## Abstract

The purpose of our study was to explore potential characteristic biomarkers in patients with sepsis. Peripheral blood specimens from sepsis patients and normal human volunteers were processed by liquid chromatography-mass spectrometry-based analysis. Outlier data were excluded by principal component analysis and orthogonal partial least squares-discriminant analysis using the metabolomics R software package metaX and MetaboAnalyst 5.0 (https://www.metaboanalyst.ca/home.xhtml) online analysis software, and differential metabolite counts were identified by using volcano and heatmaps. The obtained differential metabolites were combined with KEGG (Kyoto Gene and Kyoto Encyclopedia) analysis to screen out potential core differential metabolites, and ROC curves were drawn to analyze the changes in serum metabolites in sepsis patients and to explore the potential value of the metabolites in the diagnosis of sepsis patients. By metabolomic analysis, nine differential metabolites were screened for their significance in guiding the diagnosis and differential diagnosis of sepsis namely: 3-phenyl lactic acid, N-phenylacetylglutamine, phenylethylamine, traumatin, xanthine, methyl jasmonate, indole, l-tryptophan and 1107116. In this study, nine metabolites were finally screened based on metabolomic analysis and used as potential characteristic biomarkers for the diagnosis of sepsis.

## Introduction

Sepsis should be defined as life-threatening organ dysfunction caused by a dysregulated host response to infection^1^. In 2017, an estimated 489,000 (95% uncertainty interval [UI] 38.9–62.9) sepsis cases and 110,000 (10-1-12-0) sepsis-associated deaths were reported worldwide, accounting for 19.7% (18.2–21.4) of all deaths worldwide. From 1990 to 2017, age-standardized sepsis incidence decreased 37% (95% UI 11.8–54.5), and mortality decreased 52.8% (47.7–57.5). Morbidity and mortality from sepsis vary across regions, with the highest burden in sub-Saharan Africa, Oceania, South Asia, East Asia and Southeast Asia^2^. The proportion of hospital-acquired sepsis was 23.6% (95% CI 17–31.8%, range 16–36.4%) of all hospitalized sepsis cases. In the ICU, 24.4% (95% CI 16.7–34.2%, range 10.3–42.5%) of sepsis with organ dysfunction cases were acquired during the ICU stay and 48.7% (95% CI 38.3–59.3%, range 18.7–69.4%) originated in the hospital. The mortality rate of septic ICU patients with HA with organ dysfunction was 52.3% (95% CI 43.4–61.1%, range 30.1–64.6%)^3^. Due to the multiple presentations of sepsis, clinicians continue to face serious challenges in the diagnosis, treatment, and management of patients with sepsis^4^. Biomarkers play an important role in the early diagnosis and risk stratification of sepsis, guiding the use of antibiotics, severity and prognosis, and the assessment of efficacy^5^. More than 170 groups of biomarkers have been identified for the assessment of sepsis, including indicators such as PCT, CRP, TNF-α/IL-6, MCP-1, and miRNA^6–8^, however, different biomarkers play different roles in the pathophysiology of sepsis, and the misuse of certain biomarkers will lead to overdiagnosis and overuse of drugs such as antibiotics^9–11^. For example, due to the low sensitivity of blood cultures of pathogenic microorganisms, an increasing number of molecular biodiagnostic fields rely on the detection of bacterial DNA in blood to identify sepsis^12,13^. This new technique may lead to overdiagnosis of sepsis by misidentifying transient bacteremia without clinical significance and biological features^14^. There is an urgent need for a new biomarker that will help to diagnose and treat sepsis patients at an early stage.

Metabolomics is a research method to quantify all metabolites in biological systems and to find the relationship between metabolites and physiopathological changes, mainly involving differential changes in the anabolism and consumption metabolism of the organism^15^. By assessing the full range of endogenous metabolites and obtaining chemical fingerprints left by cellular processes as "instant" readings of gene function, enzyme activity and physiological status in the organism, overall metabolite measurements can reflect disease-related cellular biochemical activities^16–18^. Metabolomics provides a more accurate picture of the current metabolic state of the organism than conventional biomarkers, allowing us to stratify patients by phenomena and genotyping, rather than just by conventional parameters^19^. With the advancement of metabolomic analysis techniques, studies based on metabolomic student biomarkers are rapidly evolving, and statistical analysis methods to assess metabolomics have moved from traditional univariate to more complex multivariate^20^. Depending on the target and purpose of the study, metabolomics is distinguished between targeted and untargeted metabolomics. Targeted metabolomics is the analysis of a single or a few specific metabolites, while untargeted metabolomics is the comprehensive analysis of all metabolites. Common detection methods in metabolomics include nuclear magnetic resonance (NMR), gas chromatography-mass spectrometry (GC/MS) and liquid chromatography-mass spectrometry (LC/MS) techniques.LC–MS combined with GC–MS for comprehensive and comparative analysis of metabolites enables a more comprehensive assessment of metabolites extracted from samples and is widely used because of its high sensitivity, high specificity and reproducibility^21,22^.

The aim of this study was to elucidate the differential metabolic profiles between the sepsis group and normal controls using untargeted metabolomic analysis by liquid chromatography tandem mass spectrometry (LC–MS/MS) and then to identify potential metabolites used to differentiate between normal subjects and septic patients.

## Materials and methods

### Patient recruitment and clinical data collection

Peripheral blood specimens from sepsis patients (n = 22) and normal human volunteers (n = 10) in the Emergency Intensive Care Unit (EICU) of the Affiliated Hospital of Southwest Medical University from January 2019 to December 2019 were collected in this study under the support of a provincial-level project [Sichuan Provincial Department of Science and Technology project ("Construction of sepsis metabolome platform and screening of biomarkers", subject label number 2020JP)] in the early stage.Inclusion criteria were as follows: 1. Meeting the diagnostic criteria for sepsis 3.0 proposed by the American Society of Critical Care Medicine (SCCM) and the European Society of Intensive Care Medicine (ESICM) in 2016; 2. Aged between 16 and 75 years; 3.the subject or his or her legal representative agreed to participate in the study and signed an informed consent form. Exclusion criteria were as follows: 1. Patients younger than 16 years of age; 2. Those with a previous history of organ failure (i.e., heart failure, respiratory failure, liver failure, kidney failure, etc.); 3. Those with previous immune system disorders; 4. Those with previous hematologic disorders; and 5. Those who did not wish to participate in the study.

### Sample collection

In our study, 10 normal human peripheral blood specimens and 22 septic patients peripheral blood specimens of 5 ml each were collected and stored in the biospecimen bank of the Affiliated Hospital of Southwest Medical University at − 80 °C.

#### Ethical statement

Our study was approved by the Ethics Committee of the Affiliated Hospital of Southwest Medical University (Approval Number: KY2018029). All patients and volunteers participating in the study informed themselves or their legal representatives and obtained written informed consent, and we confirm that all studies involving human research participants during the course of the experiment were conducted in accordance with the Declaration of Helsinki.

#### Access to raw data

The datasets generated and analysed during the current study are available in the CNGB Sequence Archive (CNSA) of China National GeneBank DataBase (CNGBdb) with accession number CNP0002611 repository (https://db.cngb.org/).

### Untargeted metabolomics

In this project, liquid chromatography coupled with mass spectrometry (LC–MS/MS technique) was used for untargeted metabolomics analysis, and a high-resolution mass spectrometer Q Exactive HF (Thermo Fisher Scientific, USA) was used to improve metabolite coverage by acquiring data in both positive and negative ion modes separately. LC–MS/MS data processing was performed using compound Discoverer 3.1 (Thermo Fisher Scientific, USA) software, mainly for peak extraction, peak alignment and compound identification. The metabolomics R software package metaX^23^, MetaboAnalyst 5.0 (https://www.metaboanalyst.ca/home.xhtml) online analysis software and the Metabolome Information Analysis process were used for data preprocessing, statistical analysis and metabolite taxonomic annotation and functional annotation. Principal Component Analysis (PCA) was used to downscale the original multivariate data to analyze the groupings, trends (similarities and differences within and between sample groups), and outliers (presence of outlier samples) of the observed variables in the data set. The VIP values of the first two principal components of the OPLS-DA (Orthogonal Partial Least Squares Method-Discriminant Analysis) model were combined with the multiplicity of variance change (Fold change) obtained from the univariate analysis and the t test (Student's *t* test) results to screen for differential metabolites. The differentially metabolised compounds screened by the above methods will eventually be considered as potential characteristic biomarkers for the diagnosis of Sepsis.

### Data quality control

**Figure 1:**
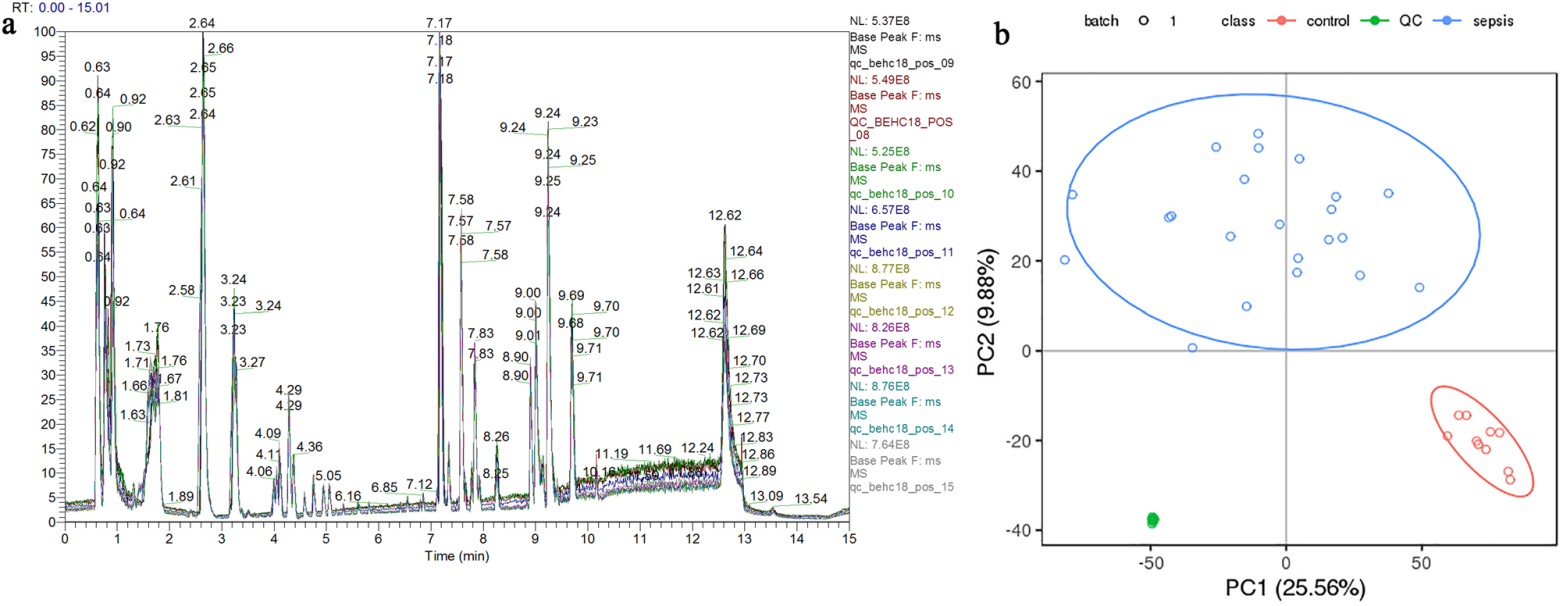
Data quality control. (**a**) Overlapping BPC (base peak ion flow diagram) plots of all QC samples. The different color peak curves in the figure represent different QC samples. All QC samples in the figure have good overlapping plots, and the retention time of each QC sample and its corresponding peak corresponding intensity fluctuations are small, which indicates that the detection instrument is operating well during the sample detection and analysis. (**b**) Principal component analysis plot. The horizontal coordinate is the first principal component PC1, the vertical coordinate is the second principal component PC2, and the ellipse is the 95% confidence interval; each circle point represents a sample, and different colors represent different groups (green is QC sample, red is normal group, and blue is sepsis group). All three groups have good differentiation, and the high overlap of QC samples indicates the more stable testing instrument.

Data quality was assessed by the reproducibility of QC sample assays, which mainly included chromatogram overlap in positive and negative ion mode for all QC samples (Fig. 1a), principal component analysis (PCA) (Fig. 1b), and compound counts (Table 1).

**Table 1:** Metabolite number statistics table.

Node	Node	Pos
Total ion number	7485	668
RSD < = 30% ion number	6807	652
Ratio (%)	90.94	97.6

Table 1 The ratio in the figure indicates that the number of metabolites with CV less than or equal to 30% of the relative peak area in QC samples is the ratio of all detected metabolites.

## Result

### Classification of metabolites

**Figure 2:**
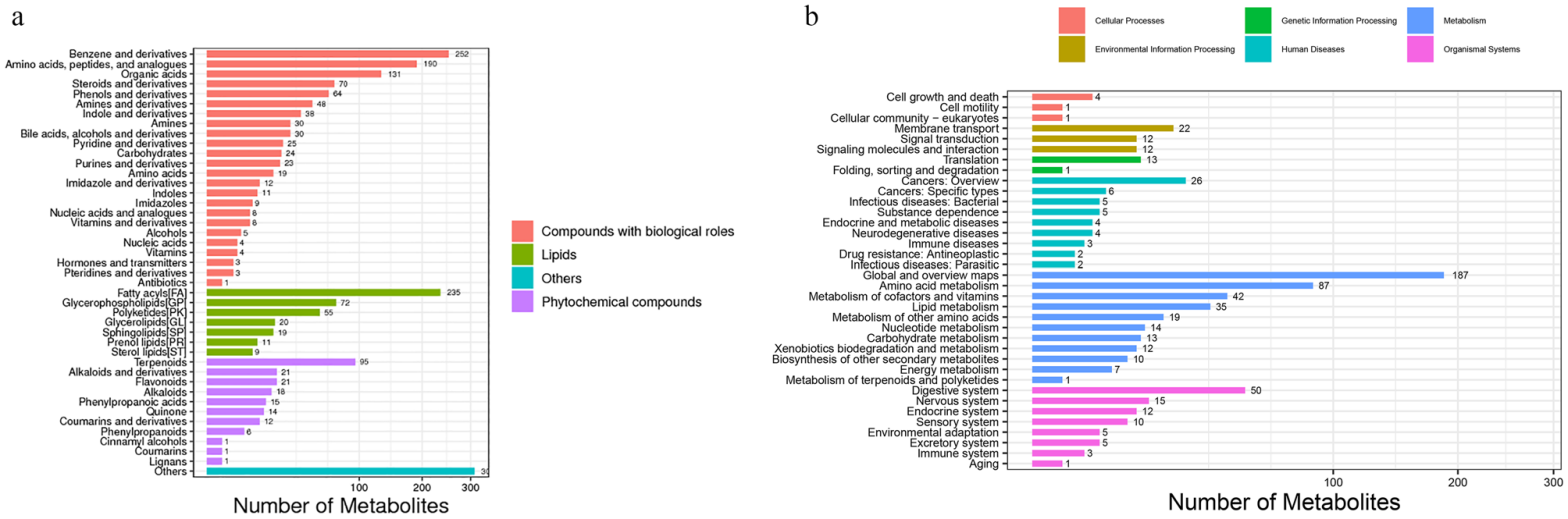
Classification of metabolites. (**a**) Metabolite classification chart. The rectangular bars of different colors in the figure represent different kinds of compounds, the X-axis represents the number of metabolite classifications, and the Y-axis represents the metabolite classification entries. (**b**) KEGG functional analysis chart. The rectangular columns of different colors in the figure represent the different types of metabolic pathways in which the metabolites are involved, with the X-axis representing the number of metabolites and the Y-axis representing the specific biological function in the metabolic pathway in which they are involved^35,36^.

The obtained specimens were thawed slowly at 4 °C, extracts were added and centrifuged several times, and the supernatants were separated and detected by LC–MS/MS technology for metabolite separation and detection. The data were processed by Compound Discoverer 3.1 (Thermo Fisher Scientific, USA) (mainly including peak extraction, retention time correction within and between groups, combined ion merging, missing value filling, background peak labeling, and data quality control), and then the molecular weight, retention time, peak area, and identification results were combined with KEGG (Kyoto Gene and Kyoto Encyclopedia), BGI Library, Chemspider database, Lipidmaps database, and HMDB database for identification, taxonomic annotation, and pathway annotation of the obtained metabolites to obtain the classification of metabolites in negative ion mode (Fig. 2a) and the metabolic pathways they are involved in (Fig. 2b).

### Screening for differential metabolites

**Figure 3:**
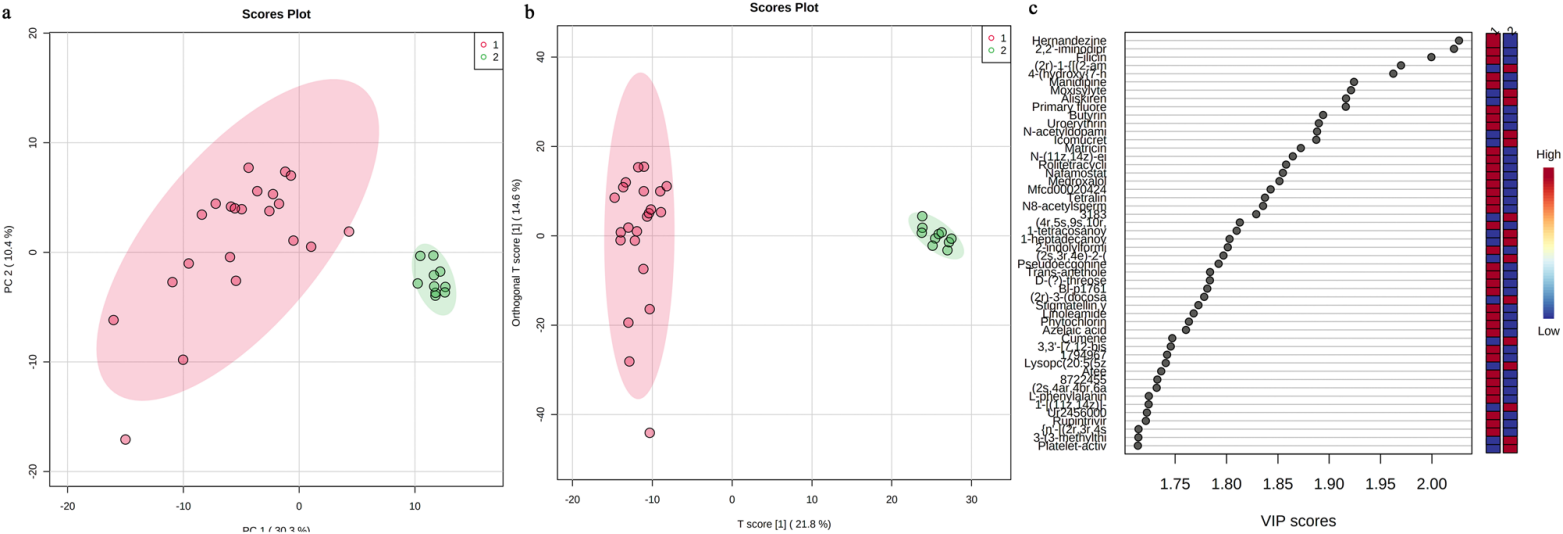
Differential metabolites screening. (**a**) PCA plot The horizontal coordinates are the first principal component PC1, the vertical coordinates are the second principal component PC2, and the ellipse is the 95% confidence interval. Each point represents a sample, and different groups are labeled with different colors (red for the normal group and blue for the sepsis group). There is good discrimination between the two groups in the figure. (**b**) Plot of the OPLC-DA analysis model The horizontal axis in the figure is the first principal component, and the vertical axis is the second principal component. The number in parentheses is the score of that principal component, which indicates the percentage of the overall variance explained by the corresponding principal component. There is a good discrimination between the two groups in the figure. (**c**) VIP value score graph The vertical coordinate in the graph indicates a metabolite, and the horizontal coordinate indicates the VIP value.

**Figure 4:**
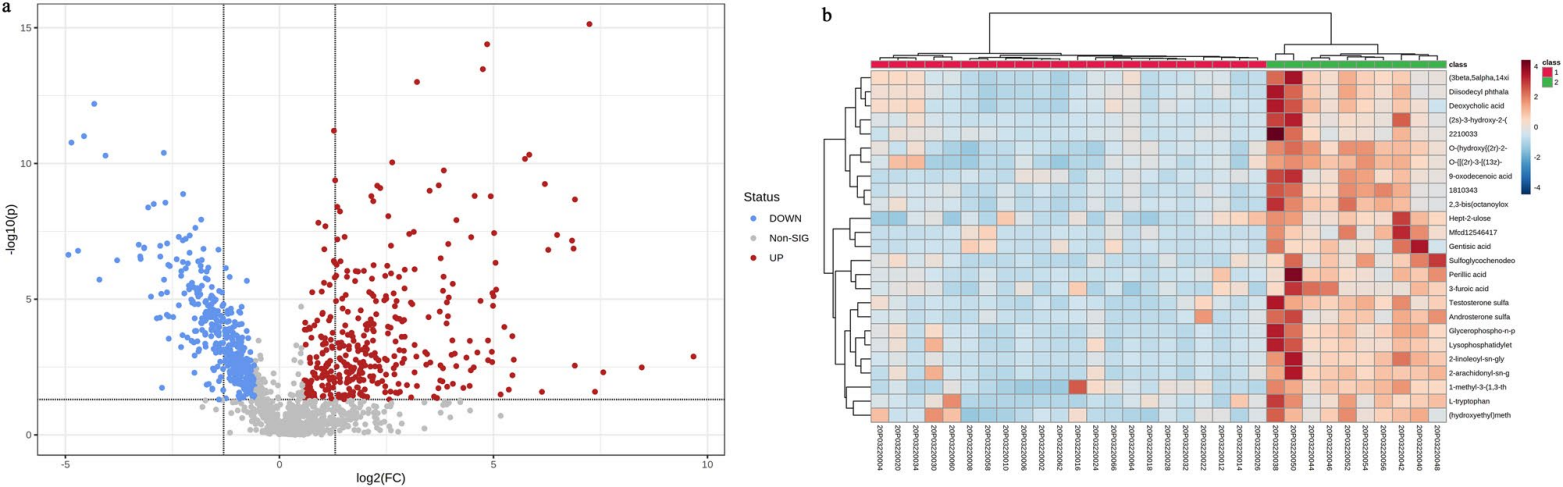
Differential metabolites screening. (**a**) Volcano plot The dots in the figure represent metabolites with VIP values greater than or equal to 1, the circles represent metabolites with VIP values less than 1, green represent downregulated metabolites, red represent up-regulated metabolites, and gray represent meaningless metabolites. (**b**) Heatmap Each column in the figure represents a sample, where group 1 is the sepsis group and group 2 is the normal control group; each row indicates a differential metabolite expression value; blue indicates downregulation and brown indicates upregulation. Made by MetaboAnalyst 5.0. (https://www.metaboanalyst.ca/home.xhtml) online analysis software.

In the two groups of samples, after normalizing the data in both positive and negative ion modes, a total of 7459 metabolites were detected, which were validated by performing principal component analysis (PCA, Fig. 3a) on the metabolites while applying orthogonal partial least squares-discriminant analysis (OPLS-DA, Fig. 3b), which was validated by OPLS-DA. The differences between the two groups of samples were quite significant, and the samples were basically in the 95% confidence interval (CI). We were also able to obtain the value of the variable impact importance factor (VIP) of the first component in OPLS-DA. This summarizes the contribution of each variable to the model, and we considered metabolites with VIP > 1 and *p* < 0.05 as metabolites with significant differences, where the VIP values of the first 50 metabolites are shown in Fig. 3c. We submitted the raw data to MetaboAnalyst online analysis software (https://www.metaboanalyst.ca/faces/home.xhtml) and obtained a volcano plot (Fig. 4a) and a visual heat map of the top 25 differential metabolites (Fig. 4b). According to the volcano plot, it can be concluded that the sepsis group had 370 high metabolites and 386 low metabolites compared to the normal group. The differential metabolites obtained by the above analysis often have similar and complementary functions biological, or are positively or negatively regulated by the same metabolic pathway and show similar or opposite expression characteristics between the sepsis and normal groups, and we applied MetaboAnalyst 5.0 software to classify the above metabolites into the organic heterocyclic compound class, organic acid class, organic carbohydrates, organic hydroxides, nucleic acids, benzenes, sterol lipids, organohalogen compounds, isoprenoids, polyketides, and fatty acyl groups.

### Metabolomics pathway analysis

**Figure 5:**
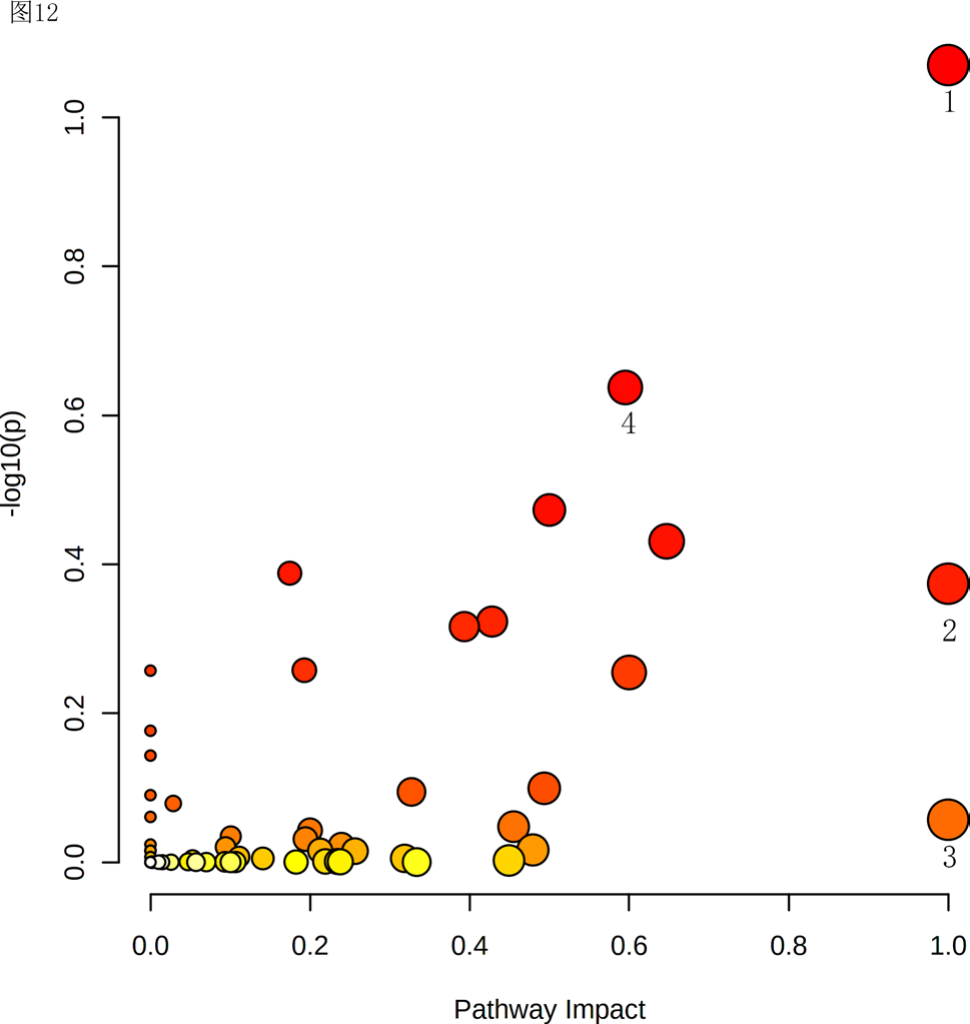
Pathway analysis. The horizontal coordinate indicates the pathway impact value, the vertical coordinate indicates the −log10(*p*) of the pathway, a dot in the figure represents a metabolic pathway, the size of the dot is proportional to its impact value, and the color of the dot represents the size of the pathway *P* value, where the color change from yellow to red represents the change in the *P* value from large to small.

The metabolites were analyzed by MetaboAnalyst 5.0 online software for metabolic pathways, and the general overview of metabolic pathways is shown in Fig. 5, while potential differential metabolic pathways were screened by combining the KEGG database with effect values > 0.5 and *P* values < 0.05. A total of four differential metabolic pathways were screened, namely, caffeine metabolism, biosynthesis of phenylalanine, tyrosine and tryptophan. Linolenic acid metabolism and phenylalanine metabolism (Supplementary Information). Combined with the previous volcano and heatmaps, the above four metabolic pathways were screened again for differential metabolites, and a total of 12 differential compounds were screened, namely, 3-phenyl lactate, N-phenylacetylglutamine, phenylethylamine, traumatin, xanthine, methyl jasmonate, indole, levotryptophan, 1107116, traumatic acid, theobromine, and salicylic acid.

### The diagnostic value of different metabolites in sepsis

**Figure 6:**
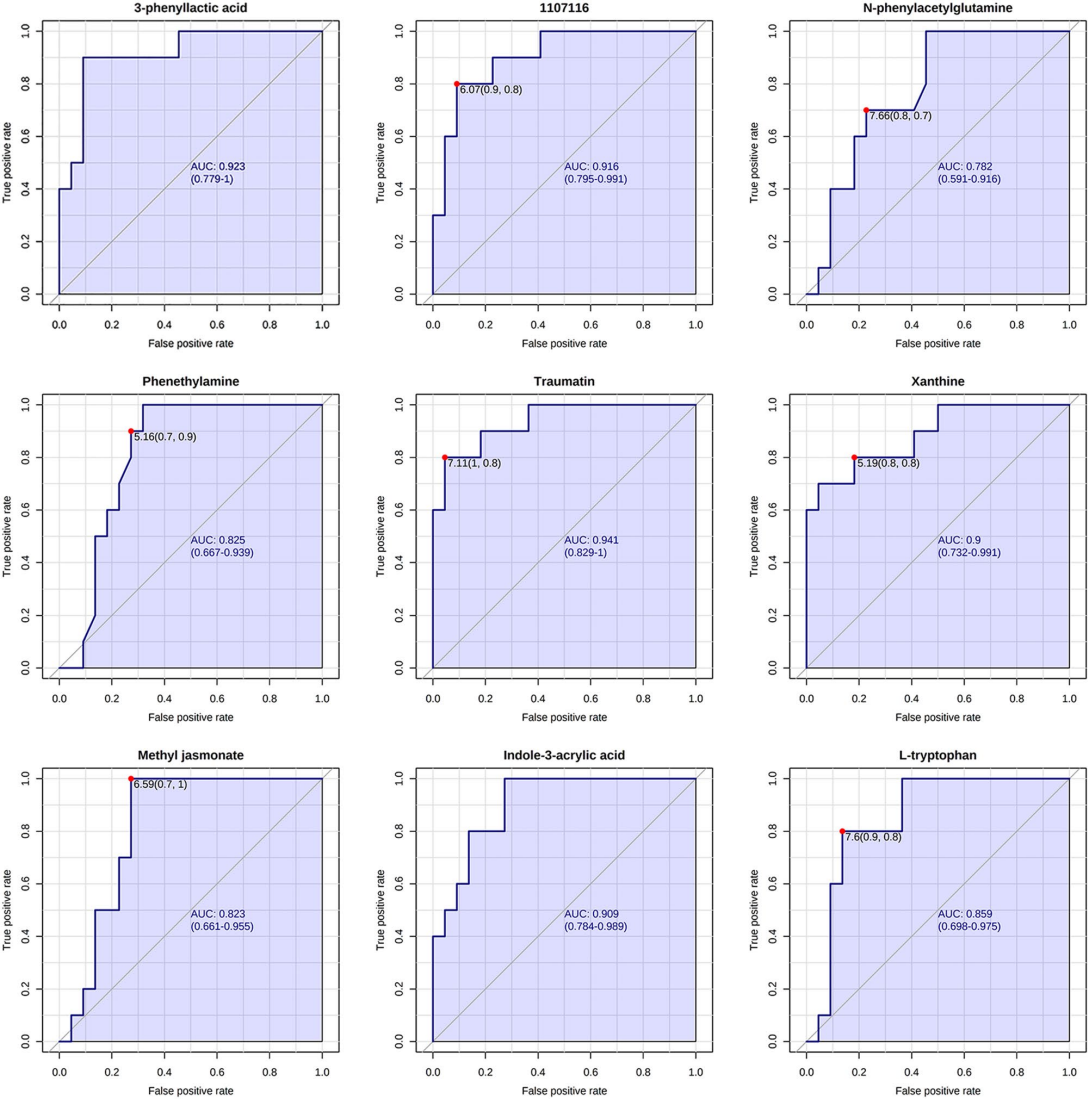
ROC curve graph. The horizontal coordinates in the graph indicate the negative positive class rate, i.e. the false positive rate (1-specificity); the vertical coordinates indicate the true class rate, i.e. the true positive rate (sensitivity), and the AUC value, i.e., the area under the curve, the higher the AUC value, i.e., The larger the area under the curve is, the higher the prediction accuracy. The closer the curve is to the upper left corner (the smaller the X and the larger the Y), the higher the prediction accuracy.

To determine the diagnostic efficiency of the above 12 potentially differential metabolites, we generated subject operating characteristic curves (ROC curves) and calculated the area under the curve (AUC) for each incorporated characteristic, according to the Swets judgment criteria^24^. AUC < 0.5 indicates that the test has no diagnostic value, AUC of 0.8–0.9 indicates that the test has good accuracy, and AUC > 0.9 indicates that the diagnostic test has high accuracy. Finally, we obtained nine differential metabolites whose expression values were significantly different between sepsis and normal control species, namely, 3-phenyl lactate (AUC: 0.923), N-phenylacetylglutamine (AUC: 0.782), phenylethylamine (AUC: 0.825), traumatin (AUC: 0.941), xanthine (AUC: 0.900), methyl jasmonate (AUC: 0.823), indole (AUC: 0.909), levotryptophan (AUC: 0.859), and 1107116 (AUC: 0.916), whose ROC curves are shown in Fig. 6.

## Discussion

The main difficulty in the diagnosis and treatment of sepsis is that its pathogenesis is not completely clear. Previous studies on sepsis mainly focus on genomics and transcriptomics. Due to the lack of specific clinical indicators for the diagnosis of sepsis, the mortality rate of this disease is very high. It has been shown that when sepsis occurs, the body is in a hypermetabolic state, and the three major nutrients, sugar, protein and lipids, all undergo constitutive changes in the body^25^, and a single biomarker is not ideal for diagnosing and judging the prognosis of sepsis. In this study, 12 differential metabolites were screened by metabolomics analysis, and 9 differential metabolites with high diagnostic efficiency were finally obtained by ROC curve analysis of the differential metabolites, including 3-phenyllactic acid, N-phenylacetylglutamine, phenylethylamine, tronin, xanthine, methyl jasmonate, indole, L-tryptophan, 1107116. Through in-depth study of these differential metabolites, we are also expected to screen out potential therapeutic targets and provide clues for targeted treatment of sepsis.

It has been shown that 3-phenyl lactate activates the NF-κB signaling pathway and triggers nuclear translocation of NF-κB causing downregulation of E6 and E7 protein levels, while increasing matrix metalloproteinase-9 (MMP-9) expression through PKC signaling phosphorylation of IKK-β, ultimately promoting cell migration and invasion in cervical cancer^26^. The analysis of metabolic pathways in this study suggests that 3-phenyllactate is mainly involved in the metabolism of phenylalanine and is highly expressed in patients with sepsis. Acetylglutamine, as a uremic solute, can contribute to cardiovascular disease in renal insufficiency^27^, and phenylacetylglutamine is elevated in the plasma of patients with early diabetic nephropathy compared to patients with normal renal function, suggesting that uremic solutes and oxidative stress markers are compounds that indicate early renal function decline in diabetic patients^28^. Our study found that N-phenylacetylglutamine is mainly involved in the metabolism of phenylalanine in the human body, and it is highly expressed in the sepsis group. Recently, it has been suggested that phenylethylamine-induced hyperthermia (PIH) activates a series of events that may lead to rhabdomyolysis, coagulation disorders, and even death^29^. Through metabolomic pathway analysis, we found that phenylethylamine was highly expressed in sepsis patients through phenylalanine metabolic pathway. Previous reports have shown that the activity of traumatic acid is similar to that of unsaturated fatty acids, with antioxidant and stimulatory effects on collagen biosynthesis^30^, and that methyl jasmonate has functions in the regulation of antioxidant defense, inflammatory biomarkers, neurotransmitter regulation, and neuronal regeneration^31^. Our study found that both wound acid and methyl jasmonate were low expressed in sepsis patients and both were involved in linolenic acid metabolism. Indole is known to be a bacterial metabolite of tryptophan, which has been proposed as a key metabolite in the regulation of inflammation, metabolism and behavior, and recently, it has been reported that indole reduces hepatic expression of inflammation-related genes, macrophage activation and markers of liver injury^32^. The latest experimental studies show that indole-3-propionic acid treatment ameliorated the middle cerebral artery occlusion-induced alterations of the gut microbiome structure, specifically reshaping the microbial community composition in mice with middle cerebral artery occlusion to resemble that in the mice from the control group, with an increase in the abundance of probiotics and a decrease in the abundance of harmful bacteria. Indole-3-propionic acid repaired the integrity of the intestinal barrier and regulated the activities of regulatory T cells (Tregs) and Th17 cells in the gut-associated lymphoid tissue^33^. In our study, by analyzing the biosynthesis process of phenylalanine, tyrosine and tryptophan, it was found that indole was at a low expression level in patients with sepsis. Recent studies have shown that in vivo tryptophan triggers a cytoprotective gene expression program by inhibiting iron death through direct scavenging of free radicals and activation of an antioxidant gene expression program after the production of indole-3-pyruvate, thereby inhibiting redox death of iron death^34^. The present study found that the expression level of L-tryptophan was low in patients with sepsis.

In our study, metabolomics analysis combined with ROC curve analysis was used to screen the differential metabolites closely related to the clinical manifestations of sepsis, so as to provide clues for targeted therapy of sepsis. However, the deficiency of the study is more apparent, we failed to sepsis patients thoroughly clarifying the process of metabolism, metabolic pathways of disease condition and signaling pathways are still not entirely clear, how the differences metabolites of selected above mediated the body inflammation reaction and the disease of which represent meaning still need further in-depth study.

## Supplementary Information

Supplementary Information.
