# Supplementary material for: Metabolomics-based study of potential biomarkers of sepsis
Source: Sci Rep. 2023 Jan 11;13:585. doi: 10.1038/s41598-022-24878-z (PMC9834301; doi:10.1038/s41598-022-24878-z)
Supplement: Supplementary file 1 — Supplementary Information. [file 41598_2022_24878_MOESM1_ESM.docx]

**Supplementary Table S1. Table of diffierentinal metabolic pathways**

| Nmuber | X.Pathway | Count | Count.All | Pvalue | Pathway.ID | Up | Down |
| --- | --- | --- | --- | --- | --- | --- | --- |
| 1 | Caffeine metabolism | 3 | 22 | 0.001279439 | map00232 | Xanthine,  Xanthosine | Theobromine |
| 2 | Phenylalanine, tyrosine and tryptophan biosynthesis | 2 | 35 | 0.0468496 | map00400 |  | Indole,  L-tryptophan |
| 3 | Alpha-Linolenic acid metabolism | 3 | 44 | 0.009384945 | map00592 |  | Traumatin,  Methyl jasmonate,  Traumatic acid |
| 4 | Phenylalanine metabolism | 6 | 72 | 0.005664142 | map00360 | 1107116,   1. acetyl-l-phenylalanine,   Phenethylamine,   1. phenyllactic acid,   N-phenylacetylglutamine | Salicylic acid |
